# Breast size, thoracic kyphosis, and thoracic spine pain: a correlational survey of Nigerian postpartum mothers

**DOI:** 10.1186/s12891-024-07978-x

**Published:** 2024-11-20

**Authors:** Ojukwu Chidiebele Petronilla, Edeani Pamela Chinecherem, Ede Stephen Sunday

**Affiliations:** 1 Department of Medical Rehabilitation, College of Medicine, University of Nigeria Enugu Campus, Preston, Nigeria; 2 School of Sports and Health Sciences, University of Central Lancashire, Preston, UK

**Keywords:** Breast size, Thoracic kyphosis, Thoracic spine pain, Postpartum women, Nigeria

## Abstract

**Background:**

Thoracic spine postural dysfunctions are common postpartum-related health problems, compromising breastfeeding efficacy and quality of life among women. Previous studies have particularly associated these conditions with increased breast sizes in several populations. However, such empirical evidence is scarce in the Nigerian population.

**Objectives:**

To investigate the relationship among breast size, thoracic-kyphosis, and -spine pain among postpartum Nigerian women.

**Methods:**

This correlational survey involved 400 consenting postpartum mothers (between 0 and 24 months of postpartum period). Their breast size, thoracic spine posture, and pain were measured using a measuring tape (cm), inclinometer, and Revised Oswestry thoracic spine pain disability questionnaire, respectively. Data were analyzed using descriptive and relevant inferential statistics at *p* < 0.05.

**Results:**

The majority of the participants fall under the category of breast cup size B (61.75%), have no history of thoracic spine pain (87.4%), and about half of them (50.2%) have normal thoracic spine posture (low category with values ranging between 20⁰ and 35⁰. Breast size was significantly (*r* = 0.162, *p* = 0.001) correlated with thoracic spine posture but showed no significant correlation (*r* = 0.066, *p* = 0.622) with thoracic spine pain.

**Conclusion:**

Increasing breast size is weakly associated with a tendency towards a kyphotic posture of the thoracic spine. Postural education and care around adequate support of the breast with suitable fitting brassieres may help prevent kyphotic deformities. Future research with a randomized control trial and long-term follow-up is recommended to further confirm the causal relationship of these variables.

## Introduction

The breast tissue undergoes physiologically mediated anatomical changes during pregnancy and lactation that is caused by Estrogen and Progesterone, which are important to prepare the breast for lactation during pregnancy [1]. Estrogen stimulates the growth of breast duct cells and the secretion of prolactin. Prolactin also stimulates breast engorgement and the production of milk. As well, the sucking action of the infant’s mouth on the breast stimulates more demand for milk, with further breast engorgement in postpartum periods [2].

These physiological changes and postpartum lactation activities all lead to possibilities for a gain in breast size and structure, which can alter the body’s biomechanics. For instance, pregnancy-related abdominal protrusion and breast enlargement result in an anterior shift in the centre of gravity and gravitational line [3]. In compensation, the body makes some adjustments to return the position of the gravitational line closer to the centre of the body’s base of support, with resultant untoward postural adjustments [4, 5]. As such, pregnant women commonly report distorted spinal alignments [6].

On the other hand, awkward positions adopted during childcare tasks including, breastfeeding [7] and infant carrying [8] may further lead to postural alterations. These postures may increase pressure on the intervertebral discs, causing strain on the back muscles, which could further lead to fatigue and discomfort [9], and consequent deformities of the thoracic spine may further ensue. Breast size increments in two or three folds are also evident during lactation [1], resulting in tendencies for women to wear bigger fitting brassieres during their breastfeeding periods [10]. Spinal inclination most likely increases in the presence of a heavy load on the anterior thoracic wall [11]. Since the breast exerts an anterior mass effect, there is a likelihood for a gravity-influenced downward drag of the breast weight with resultant increments in thoracic kyphosis and cervical lordosis, generating alterations in the centre of gravity with additional tension experienced during cervical extension [12]. The discomfort experienced by the downward drag would be estimated by breast mass and other cutaneous tissues, implying that overall body weight and Body Mass Index (BMI) may play a role in the symptom route [13]. Findikcioglu et al. [14] postulate that the effects of breast size on spinal posture vary with breast size, bringing about biomechanical claims for large breast symptoms.

Correlations among breast size, thoracic kyphosis, and thoracic spine pain have been reported in several populations in previous studies [13–17]. For instance, Coltman et al. [17] carried out a quantitative study among 378 women aged 18 + years to examine if breast characteristics predict upper torso musculoskeletal pain. Their finding demonstrated that breast volume, age, and nipple-to-nipple distance predicted 23% of the variance in the upper torso of musculoskeletal pain. In another population of post-menopausal women, increased breast size and BMI were associated with thoracic pain without any influence on thoracic spine posture. Spencer and Briffa [13] reported pain in the scapular elevator muscles (T7/T8) induced by a downward drag of the breast weight in women with large breasts who wore poor-fitting brassieres. However, an older study [15] showed no association between breast size and thoracic spine pain in young nulliparous women.

Considering that the postpartum period is associated with changes in breast structure and orientation, it is possible that these correlations will exist during the postpartum period but such studies are limited in the breastfeeding population, particularly in African clime. There is a need to explore such relationships to enable easy identification of nursing mothers at risk of thoracic spine dysfunctions. Thus, this study aimed to explore possible relationships among breast size, thoracic spine posture, and pain in a population of Nigerian postpartum mothers.

## Methods

### Study participants

This cross-sectional correlational survey involved 400 postpartum mothers within 0 to 24 months post-delivery duration who were conveniently recruited from the postnatal clinics of four selected hospitals in Enugu, Nigeria. The exclusion criteria included women who have undergone previous breast transplants, had previous diagnosis thoracic spine deformities (postural kyphosis, Scheuermann’s kyphosis, congenital kyphosis, lordosis, and scoliosis) before pregnancy, currently have breast mastitis as well as complications from spinal anesthesia administered during previous caesarean sections. The sample size was estimated using Cohran [18] formula since the precise population size of postpartum women in the area of study is unknown. The error allowance was set at 0.05, and the Z score for the confidence interval was set at 1.96 based on previous studies on the prevalence rate (28.9%) of thoracic spine pain in Nigerian pregnant women [19, 20]. Thus, the result of the estimated sample size was 300.

### Study instrument

Participants’ breast size, thoracic spine posture, and thoracic spine pain were measured using measuring tape (cm), inclinometer, and Revised Oswestry thoracic spine pain disability questionnaire, respectively.

The breast size was measured based on the Triumph International size chart, which is a brassieres sizing chart that contains the under-bust circumference in centimeters (cm) and the difference between the under-bust and over-bust which is graded in letters (A, B, C, D) [15]. Breast mass, breast size score, cup size, and brassieres size are different measures of estimating breast size [13]. Cup size indicates a woman’s breast size, but it can be relative to the body size of the woman while breast mass and breast size score are independent of the body size of the woman [13]. Therefore, breast mass can be more appropriate for biomechanical studies and was chosen for this study. The cup size can be estimated by comparing the under-bust (circumference of the chest below the breast) and the bust circumference (mostly taken at the nipple landmark) [21]. It is represented with the alphabets A, B, C, and D. The bra size is estimated by the difference between under-bust and over-bust circumference. A difference of 2.54 cm (1 inch) is the equivalent of an A-cup size, 5.08 cm (2 inches) is a B-cup size and 7.62 cm (3 inches) is a D-cup size [22]. It is recommended that women with a cup size ≥ D or bra size ≥ 18 could be categorized as having large breasts [23]. The reliability for this approach of measuring breast size has been reported by Schinkel-Ivy and Drake [22] by assessing eight subjects who completed each of the measures three times. They reported an intraclass correlation coefficients of 0.999 and 0.995 were found for the under-bust and over-bust circumference, respectively, and the corresponding standard errors of measurement were 0.18 cm (0.22% of the participants’ mean measurements) and 0.28 cm (0.39%).

The revised Oswestry thoracic spine pain disability questionnaire consists of 10 items addressing different aspects of function. Each item is scored from 0 to 5, with higher values representing greater disability. The total score is multiplied by 2 and expressed as a percentage. These were interpreted as follows: 0–20%, minimal disability; 21–40%, moderate disability; 41–60%, severe disability; 61–80%, very severe; 81–100%, complete dependency [24]. This tool has been used and proven valid in this population [24, 25].

In addition, a self-structured questionnaire was also used to elicit data on participants’ social demographics and past and present obstetrics history of the postpartum women.

### Data collection

Under the guidance of the researchers/assistants, participants filled out the self-structured questionnaire after they provided informed consent and were given prior orientation about the study procedure. Those who had thoracic spine pain filled out the Revised Oswestry thoracic spine pain disability questionnaire.

Subsequently, one of the lead researchers (EPC) conducted all the measurements to reduce inter-rater bias; assisted by research assistants. The chest circumference measurements of each participant were taken in an excluded room for privacy purposes. These measurements were done over very light clothing without a brassiere. For each participant, two chest circumferences (under-bust and over-bust) were measured. After expiration, the under-bust chest circumference was measured at the level of the inferior mammary fold with the measuring tape kept level and tight across the back. Similarly, the over-bust chest circumference was taken after expiration with the nipple as a landmark. Breast size was calculated as over-bust circumference subtracted from the under-bust circumference and reported in inches. A difference of 2.54 cm (1 inch) is the equivalent of an A-cup size, 5.08 cm (2 inches) is a B-cup size, and 7.62 cm (3 inches) is a D-cup size [22].

For the thoracic spine posture measurements, participants were requested to remove all upper body clothing to expose the back in order to ensure accuracy. Two pencil lines were made on the skin over the spine on the C7 and T12 spinous processes. The final rib was used as the landmark to locate the T12 process and palpation was used to locate the C7 spinous process, which is thought to be the most prominent at the cervicothoracic junction [26]. Two inclinometers were used simultaneously to record the dynamic motion of the spine during spinal flexion. One inclinometer was placed at the C7 spinous process and the other at the T12 spinous process. The value of the two measurements was added to get the Cobb angle [27]. The measurements of the participants were categorized into normal (lower category) which is 20⁰-35⁰, normal (higher category) which is 36⁰-50⁰, and hyperkyphosis which is 51- above.

### Data analysis

Data were analyzed using descriptive statistics to present the socio-demographic and obstetrics characteristics of the participants. Inferential statistics of Pearson’s correlation were used to estimate the relationship among breast size, thoracic kyphosis, and thoracic spine pain. Correlation coefficients (r) of 0.00–0.19, 0.20–0.39, 0.40–0.59, 0.60–0.79, and 0.80–1.00 were considered very weak, weak, moderate, strong, and very strong, respectively [22]. All analyses were performed with a statistical package of social sciences software version 24 (SPSS inc, USA) at *p* < 0.05.

## Results

### Socio-demographic and general characteristics of participants

The study involved 400 postpartum women with an average age of 28.3 ± 5.0 years and an average BMI of 28.2 ± 5.0. The mean over-bust circumference and under-bust circumference are 35.6 ± 3.5 and 33.4 ± 15.3 respectively.

Table 1 shows that for the majority of participants (34.3%), the difference in their cup sizes ranged between 2.1 and 3.0 inches. Most of the participants are within the age range of 21–40 years (98.0%), had secondary school as their highest level of education (47.5%), are currently employed (58.5%), professionals (29.90%) and did not engage in occupations that required prolonged bending (61.0%). A greater percentage of the participants were multigravida with 2–4 pregnancies (61.3%), multiparous with 2–4 childbirths (60.8%), and had 1–4 children (92.0%).

**Table 1:** General characteristics of the postpartum mothers (*N* = 400)

Variable	Frequency	Percentage (%)
**Cup size (difference between under bust and over bust in inches)** A- (0–1)	4	1.0
B- (1.1-3)	247	61.7
C-(3.1-4)	87	21.8
D-(4.1-5)	46	11.5
E-(5.1-6)	13	3.3
F-(6.1-7)	3	0.7
**Age(years)** > 20	12	3.0
21–40	380	98.0
> 40	8	100.0
**Level of Education** Primary	16	4.0
Secondary	190	47.5
Tertiary	180	45.0
Postgraduate	14	3.5
**Employment Status** Yes	234	58.5
No	166	41.5
**Employment in the last 12 months (n = 166)** Yes	33	19.9
No	133	80.1
**Occupation (n = 234)** Professional	70	29.9
Clerical	22	9.4
Sales	56	23.9
Skilled Manual	69	29.5
Unskilled Manual	6	2.6
Agriculture	11	4.7
**Engagement in occupation that requires prolonged bending** Yes	156	39.0
No	244	61.0
**Number of pregnancies** Primigravida(1st pregnancy)	123	30.8
Multigravida(2–4 pregnancy)	245	61.3
Multigravida(> 4 pregnancy)	32	8.0
**Parity** Primiparous	125	31.3
Multiparous(2–4 childbirth)	243	60.8
Multiparous(> 4 childbirth)	32	8.0
**Number of Children** 1–4	368	92.0
**>** 4	8.0	8.0
**Number of children ever breastfed** 1 Child	142	35.6
2–4 children	226	56.6
4–10 children	32	7.8
**Current breastfeeding status** Yes	368	92.0
No	32	8
**Number of child(ren) currently being breastfed** 1	344	91.7
> 1	31	8.3
**Most commonly adopted breastfeeding position** Cradle	228	57.0
Cross cradle	66	16.5
Football hold	70	17.5
Laid- back	6	1.5
Side lying	30	7.5
**Use of breastfeeding pillows** Yes	99	24.8
No	301	75.3
**Use of Infant Carrier** Yes	151	37.8
No	249	62.2
**Commonly utilized infant carrier method with infant carriers** Back	7	4.5
Front	147	95.5
**Body weight of currently breastfed baby** 0–5 kg	21	58.3
5.1–20 kg	15	41.7
**Current practice of postnatal exercise** Yes	52	13.0
No	348	87.0
**Mode of active physical exercise** Aerobics	28	53.8
Stretches	22	42.3
Others	2	3.8
**Commonly utilized type of breastfeeding brassiere** Convertible	25	6.3
Basic barrette	42	10.5
Push up	162	40.5
T-shirt/nursing	165	41.3
Sport bra	6	1.5

Table 1 also shows that majority of the women (56.6%) have breastfed a total of 2–4 children in their histories while 92.0% were currently breastfeeding infants. A majority of the women adopted cradle position as their most common breastfeeding position (57.0%), did not use breastfeeding pillows for support during nursing (75.3%), commonly adopted front infant carrying methods (95.5% ), were not using infant carriers for infant carrying tasks (62.2%), had their current breastfeeding infant weight between 0 to5kg (58.3%), were not currently practicing postnatal exercises (87.0%) and had nursing brassiere as their commonly utilized type of breastfeeding brassiere (41.3%).

### The relationship among participants’ breast size, thoracic spine posture, and thoracic spine pain

The mean thoracic spine posture, thoracic spine pain and breast size values of the participants were 37.9 ± 7.9^0^, 4.4 ± 1.1 and 3.0 ± 1.1 respectively. The results presented in Table 2 shows that the majority of the participants fall under the category of breast cup size B (61.75%) and have no history of thoracic spine pain (87.4%). About half of them (50.2%) have normal values of thoracic spine posture (within the lower category with values ranging between 20 degrees and 35 degrees). 11 participants presented with kyphotic postures of which 4 belonged to the breast cup size B category. Pearson’s correlation analysis showed a weak significant positive correlation (*r* = 0.162, *p* = 0.001*) between the participants’ breast size and thoracic spine posture.

**Table 2:** Relationship between breast size and each of thoracic spine posture and pain intensity of the participants

Variables	Breast Cup Size							
Thoracic Spine Posture	A *n*%	B *n*(%)	C *n* (%)	D *n*(%)	E *n*(%)	F *n*(%)	Total	*R* value (*P* value)
Hypo kyphosis	0	0	0	0	0	0	0	
Normal (Low category 20°-35°)	2 (50.0)	124 (50.2)	33 (37.9)	16 (34.7)	5 (38.4)	0 (0)	180 (45)	
Normal (High category 36°-50°)	2 (50.0)	119 (48.1)	51 (58.6)	27 (58.6)	7 (58.8)	3 (100)	209( 52.2)	0.162 (0.001)*
hyper kyphosis	0 (0)	4 (1.6)	3 (3.4)	3 (6.5)	1 (7.6)	0 (0)	11 (2.7)	
Total	4 (1)	247 (61.75)	87 (21.75)	46 (11.5)	13 (3.25)	3 (0.75)	400 (100)	
**Thoracic Spine Pain Intensity**								
None	4 (100)	216 (87.4)	68 (78.1)	41 (89.1)	9 (69.2)	3 (100)	341 (85.25)	
Mild	0 (0)	9 (3.6)	6 (6.8)	1 (2.1)	1 (7.6)	0 (0)	17 (4.25)	
Moderate	0 (0)	19 (7.6)	9 (103)	4 (4.6)	2 (15.3)	0 (0)	34 ()	0.066 (0.622)
Severe	0 (0)	3 (1.2)	4 (4.5)	0 (0)	1 (7.6)	0 (0)	8 (2)	
Total	4 (1)	247 (61.75)	87 (21.75)	46 (11.5)	13 (3.25)	3 (0.75)	400 (100)	

Key: Percentages were derived from the sum of the breast size

* indicates significance at p < 0.05

Cup size A: difference of 2.54 cm (1 inch)

Cup size B: difference of 5:08 cm (2inches)

Cup size C: difference of 7.62 cm (3inches)

Cup size D: difference of 10.16 cm (4 inches)

Cup size E: difference of 12.7 cm (5 inches)

Normal low: 20°-35°

Normal high: 36°-50°

Kyphosis: 51°- above

Mild: 0–3

Moderate: 4–6

Severe: 7–10

Table 3 further shows that out of 59 participants with thoracic spine pain 31 belonged to breast cup size B category. A non-significant positive correlation (*r* = 0.066, *p* = 0.622) existed between the participants’ breast size and thoracic spine pain. On the other hand, there was no significant correlation (*r*= -0.141 and *p* = 0.285) between participants’ thoracic spine pain and thoracic spine posture.

**Table 3:** Relationship between thoracic spine posture and Pain Intensity

Variables	Thoracic Spine Pain					
Thoracic Spine posture	None *n* (%)	Mild *n* (%)	Moderate *n* (%)	Severe *n* (%)	Total	*R* value (*P* value)
Normal (Low category 20°-35°)	158(46.3)	6(3.5)	13(38.2)	3(37.5)	180(45)	
Normal (High category 36°-50°)	175(51.3)	9(52.9)	20(58.8)	5(62.5)	209(52.2)	-0.141(0.285)
Hyper kyphosis	8(2.3)	2(12.7)	1(2.9)	0(0)	11(2.7)	
Total	341(0.25)	17(4.25)	34(8.5)	8(2)	400(100)	

Key: Percentages were derived from the sum of thoracic spine pain

Normal low: 20°-35°

Normal high: 36°-50°

Kyphosis: 51°- above

Mild: 0–3

Moderate: 4–6

Severe: 7–10

## Discussion

This study is aimed at determining the relationship among breast size, thoracic kyphosis, and thoracic spine pain among postpartum women in Enugu State. The main finding showed that the majority of the participants fall under the category of breast cup size B, and breast size showed a significant positive correlation with thoracic spine posture but was not significantly correlated to thoracic spine pain. A detailed discussion of these findings is presented in the sections that follow.

### Relationship between breast size and thoracic spine posture

This study finding demonstrated the existence of a weak significant positive relationship between breast size and thoracic spine posture, which indicate that increasing breast size could be linked to an increasing kyphotic posture. This agrees with previous research findings that large breast can move the center of gravity away from the spine, and increase the amount of muscular effort needed to maintain balance [15]. In addition, increased breast size could cause a resultant gravitational pull leading to an anterior shift in the line of gravity, which could produce the downward drag effect with resulting compensatory mechanisms that could lead to postural changes in the biomechanics of the spine [14]. These changes could result to an increasing thoracic spine deformity such as thoracic kyphosis and cervical lordosis [28, 29], with an associated back pain. These finding is both consistent with the established biomechanically principles of force and balance and are consistent across identified literature on this topic. For instance, in a study done by Spencer and Briffa [13], large breasts and increased body mass index in postmenopausal women were related to thoracic kyphosis while they explained that the loaded spine-associated muscles may be affected biomechanically by increasing breast size and how a brassiere is worn. Also, in a cross-sectional study done by Spencer et al. [30] the possibility of thoracic vertebral fracture in post-menopausal women with large breast were noted due to increasing the mechanical loading of the spine, which was independent of the body mass density, age, thoracic kyphosis, and upper back extensor muscle endurance. This consistency is understandable given the high tendency of spinal stability to be distorted when slight lines of force are transmitted down the cervical column [31, 32].

Thus, considering that pregnancy and lactation are associated with increased breast size, women must be advised on thoracic spine care during these periods. Such care includes back extensor exercises to strengthen the back muscles, wearing firmly fitted brasserie to support the breasts [15], as well as adopting proper back posture while carrying out daily childcare activities and lifting heavy objects.

### Relationship between breast size and thoracic spine pain

On the other hand, this study finding did not show any significant effect between breast size and thoracic spine pain. While Wood et al. [15] had similarly reported that thoracic pain is unrelated to breast size in young women, this contradicted the initial hypothesis of this study that increased downward drag in large-breast individuals can lead to postural alterations, which will result in muscle tightness and possibly pain. Notwithstanding, some other studies have shown positive relationships between breast size and thoracic spine pain. Coltman et al. [17] reported a likelihood of upper torso musculoskeletal pain increasing with age, nipple-to-nipple distance, and breast volume in women. In postmenopausal women, Spencer and Briffa [13] also reported that large breasts and increased body mass index are related to thoracic spine pain. In addition, a recent literature review by Zielinsk et al. [33] concluded that there is considerable evidence to support that increasing breast size influences musculoskeletal pain. Besides, large breasts have been earlier reported to be accompanied by physical symptoms such as recurrent intertrigo in the inframammary folds, stiff neck, painful brassiere strap grooving, and chronic neck, shoulder, and back pain [14]. Again, large, or heavy breasts might put constant strain on the middle and lower trapezius muscle fibers and related muscle groups [34]. The difference in the present study findings could be attributed to the young age characteristics of this study population and the different approaches to measuring pain across the study. For instance, while this study utilized postpartum women with the average age of 28.3 ± 5.0 years when compared to those in the study of Spencer and Briffa [13] which is 50–84 years. More study is therefore needed to further verify the link between breast size and thoracic spine pain.

### Relationship between thoracic spine pain and thoracic kyphosis

Furthermore, this study’s findings show that an increase in thoracic spine posture such as hyper kyphosis is not associated with thoracic spine pain in postpartum women. This could be due to a remarkably low percentage of the women who had pain (only 16%). Similar studies are scarce among postpartum women except for the work of Spencer and Briffa [13] who showed in the contrarily that at the mid-thoracic level, the pain was significantly related to breast size and body weight. Since spinal muscle activation can affect postural alterations, it makes sense that it could affect back pain and mobility since the biomechanical significance for the loaded thoracic spine is influenced by the increasing breast size and how a bra is fitted [35]. The difference in findings observed may be because the analysis here only considered kyphosis and pain and due to the majority of the women had normal thoracic spine posture values which are not expected to elicit pain. Among those who showed kyphotic postures, they were uncertain about the duration of the deformities and if they were permanent. This study could not ascertain the duration of such postural deformities as it utilized the cross-sectional methodology. Long-standing deformities will possibly elicit more untoward effects, including pain as seen in other available studies that assessed such relationships in other populations and showed contradicting findings [36, 37]. For instance, Ryan et al. [36] reported that thoracic kyphosis is related to thoracic spine pain among osteoporotic patients while Ensrud et al. [37] showed that thoracic kyphosis is associated with interscapular pain in post-menopausal women.

### Strength and limitations

This study’s strength is in its novel contribution to this topic for the studied population where there was paucity of data in the literature. More so, the study utilized researcher and laboratory-controlled observatory measures to allow for good reliability of results obtained across the participants. However, some limitations in this study are worthy of note including those inherent in the use of the inclinometer for kyphosis assessment as their reliability can vary depending on the operator’s experience and consistency [38], as well as the participant’s varying body type, posture, and positioning [39]. These limitations of the inclinometer might have influenced the results of this study compared to studies assessing the angle of Cobb with more advanced tools like the X-ray. Also, the cross-sectional nature of this study means there is a poor estimate of causality and future studies are recommended for a robust study methodology to further confirm the findings of this study.

## Conclusion

This study shows that increasing breast size is weakly associated with a tendency towards a kyphotic posture of the thoracic spine. Despite the weak correlation, postural education and care around adequate support of the breast with suitable fitting brassieres may prevent kyphotic deformities. Future research with a randomized control trial and long-term follow-up is recommended to further confirm the causal relationship of these variables.

## Data Availability

The key data supporting the findings in the study have been provided in tables. The raw data are not publicly available due to ethical restrictions but can be obtained from the corresponding author upon reasonable request.
